# Characterising user engagement with mHealth for chronic disease self-management and impact on machine learning performance

**DOI:** 10.1038/s41746-024-01063-2

**Published:** 2024-03-12

**Authors:** Christopher Duckworth, Bethany Cliffe, Brian Pickering, Ben Ainsworth, Alison Blythin, Adam Kirk, Thomas M. A. Wilkinson, Michael J. Boniface

**Affiliations:** 1 IT Innovation Centre, Digital Health and Biomedical Engineering, School of Engineering, University of Southampton, Southampton, UK; 2 School of Psychology, Faculty of Environmental and Life Sciences, University of Southampton, Southampton, UK; 3 my mHealth Limited, London, UK; 4 National Institute for Health Research Biomedical Research Centre, University of Southampton, Southampton, UK; 5 Faculty of Medicine, University of Southampton, Southampton, UK

**Keywords:** Respiratory tract diseases, Behavioural methods, Machine learning

## Abstract

Mobile Health (mHealth) has the potential to be transformative in the management of chronic conditions. Machine learning can leverage self-reported data collected with apps to predict periods of increased health risk, alert users, and signpost interventions. Despite this, mHealth must balance the treatment burden of frequent self-reporting and predictive performance and safety. Here we report how user engagement with a widely used and clinically validated mHealth app, myCOPD (designed for the self-management of Chronic Obstructive Pulmonary Disease), directly impacts the performance of a machine learning model predicting an acute worsening of condition (i.e., exacerbations). We classify how users typically engage with myCOPD, finding that 60.3% of users engage frequently, however, less frequent users can show transitional engagement (18.4%), becoming more engaged immediately ( < 21 days) before exacerbating. Machine learning performed better for users who engaged the most, however, this performance decrease can be mostly offset for less frequent users who engage more near exacerbation. We conduct interviews and focus groups with myCOPD users, highlighting digital diaries and disease acuity as key factors for engagement. Users of mHealth can feel overburdened when self-reporting data necessary for predictive modelling and confidence of recognising exacerbations is a significant barrier to accurate self-reported data. We demonstrate that users of mHealth should be encouraged to engage when they notice changes to their condition (rather than clinically defined symptoms) to achieve data that is still predictive for machine learning, while reducing the likelihood of disengagement through desensitisation.

## Introduction

Chronic diseases are the leading cause of death and disability worldwide and represent 75% of the cost of healthcare^1,2^. As well as long-term care plans (with adherence crucial for health outcomes, quality of life and minimising healthcare cost) effective management needs the active participation of patients. Chronic diseases, however, by nature are long-term and carry a psychological burden for individuals aiming to continually manage their condition effectively^3,4^. Technological advancements in mobile Health (mHealth), healthcare and public health practice supported by mobile devices and websites can help streamline care and provide resources to reduce disease burden. Over 2.5 billion people own a mobile device worldwide highlighting the huge potential for mHealth to facilitate access to effective care^5^.

mHealth apps have the potential to be powerful platforms for positive behavioural change; both for individuals independently monitoring their health (e.g. smart watches) and for encouraging effective management of chronic disease through clinically established prevention and treatment strategies^6^. App function can range from symptom and medication diaries, educational resources, to the gamification of self-management^7^. mHealth apps also have the potential to provide early-warnings of increased risk of poor outcomes from chronic diseases (i.e., Just-in-Time Adaptive Interventions (JITAI)) by making use of clinical data in tandem with self-reported data captured in-app^8,9^. JITAIs can leverage the data collected in these apps to increase the personalisation of care and ensure treatment is provided in a timely manner through the provision of models involving machine learning (ML)^10^. As with clinical treatment, the effectiveness of mHealth is reliant on the continued engagement of users^11^. The safety of machine learning models designed to provide early risk warnings depend on data that has sufficient predictive value and quality. Depending on self-reported data raises the concern that data collection introduces additional self-management treatment burden for users. In the design of mHealth apps, there is a clear need to balance the benefit of prediction against the treatment burden of in self-reporting.

We focussed on a widely used and clinically validated mHealth app; myCOPD^12–14^ which is designed for the self-management of Chronic Obstructive Pulmonary Disease (COPD). COPD is a common, costly, and incurable respiratory disease predicted to be the third most common cause of death by 2030^15^. A key characteristic of managing COPD is mitigating the risk of ‘exacerbations’, defined by an acute worsening of a patient’s condition requiring a change in medication or emergency assistance^16^. myCOPD is provided to users diagnosed with COPD by clinicians as an explicit and agreed part of their long-term management plan.

The purpose of this research is to explore how user engagement with mHealth apps impacts predictive machine learning using self-reported data, and to discuss implications for balancing safety and treatment burden in mHealth design and engineering. To achieve this, we classified how myCOPD users engaged with the app around an exacerbation and quantified how engagement and data quality impacts the performance and safety of a ML model predicting risk to health (i.e., exacerbations). We supported this with focus group discussions and semi-structured interviews with myCOPD users to identify challenges facing digital approaches using predictive models and highlight factors leading to increased engagement and more insightful data.

## Results

### App usage and engagement

**Fig. 1:**
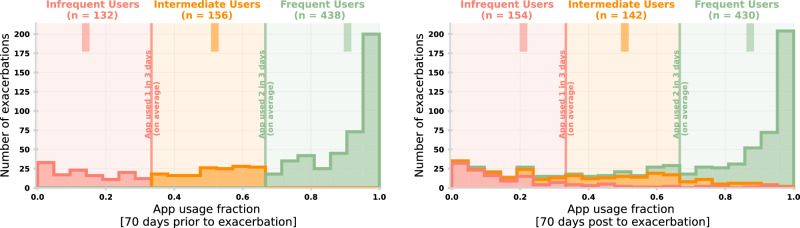
Distribution of app usage around exacerbations. (Left) The distribution of app usage in the 70-days prior to an exacerbation. This distribution is subdivided into infrequent (red), intermediate (orange) and frequent (green) users. These are separated by app usage one-in-three days on average and two-out-of-three days on average. The mean app usage fraction of each group is given by the dark shaded line segment at the top. (Right) The distribution of app usage in the 70-days post exacerbation. The original classifications from the left panel are maintained and the histograms are stacked to show overall distribution. The top annotation shows the total number of users in each classification for that given period.

App usage was quantified by the fraction of days that the user was active (i.e., registered a symptom score) out of the 70-days prior to an exacerbation. A 70-day window was chosen empirically to be long enough to define the user’s typical engagement with the app while still demonstrating trends linked to exacerbation. In myCOPD, a symptom score must be registered before accessing further app functionality (on the first opening per day). A registered symptom score therefore represents a 1-to-1 relationship with app use on a given day. Figure 1 (left) shows the distribution of app usage prior to the 727 registered exacerbations. App usage is divided into three groups: frequent users (green, *N* = 438) who register app activity ≥66% of the possible days, intermediate users (orange, *N* = 156) who use the app between 33% and 66% of possible days, and infrequent users (red, *N* = 132) who are active <33% of possible days.

Reasons for engagement were explored in semi-structured interviews. Despite some participants noting limited use of the app, most found it helpful for logging their medication use and acting as a reminder to take medications regularly. Participants also noted that the app was a source of education around self-management, which motivated engagement.

*“I used to take my medicine at all different times, and now I use it at the same time every day. And the breathing exercises and how to clear your chest and that, I didn’t know any of that before I started using the app so that’s been a great help.” [P7–male]*

A further motivator was the opportunity the app offers to monitor symptoms, which provides reassurance that they are not deteriorating.

*“I do like to look back when I’ve done the COPD assessment test, am I getting worse, am I getting better, and the answer is usually ‘no, you’re just the same’. It’s a bit of a comfort thing to have around.” [P4–male]*

This is also evidenced by in-app data with over 60% of in-app interactions being related to medication or symptom monitoring.

### Self-reported data quality and transitional engagement

**Fig. 2:**
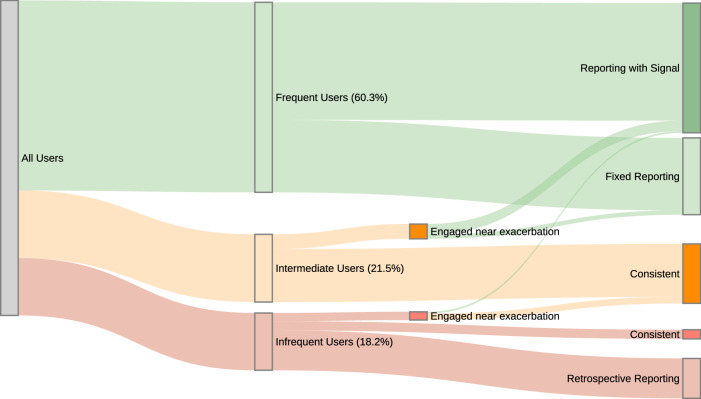
Schematic of users grouped by engagement and data quality for self-reported symptom scores prior to exacerbation. Vertical segments are proportional to the size of the group. Users are first divided by 70-day app use (as in Fig. 1) before subdivision based on self-reporting data quality (i.e., reporting with signal, fixed reporting) and transitional engagement (i.e., engaged near exacerbation). Groups chosen to investigate in Fig. 3 are darker shaded.

Figure 2 provides a schematic of user groups divided by engagement (as in Fig. 1) and self-reported data quality prior to an exacerbation. The size of each vertical segment is proportional to the size of the group. Engagement and data quality is characterised by self-reported symptom scores.

Frequent users provide self-reports with a range of data quality (i.e., use for predictive models). ‘Reporting with Signal’ corresponds to users who show sufficient variability in their self-reports that the deterioration in condition is clear leading up to the exacerbation (i.e., gradually reporting higher scores). Conversely ‘Fixed Reporting’ corresponds to users who register consistently low or high scores (i.e., only 1 s or 3 s) prior to the exacerbation. Similar proportions of reporting with signal are found for intermediate and infrequent user groups.

Intermediate and infrequent user groups can ‘transition’ to become more engaged closer to exacerbation. We find 21.8% of intermediate and 14.4% of infrequent users (classification based on 70 days prior) transition to increased engagement groups in the 21 days immediately prior to the exacerbation (i.e., ‘Engaged Near Exacerbation’). We note that most infrequent users (69.7%) are ‘Retrospective Reporting’ a rescue pack, registering the medications in-app more than 10 days after the event and providing minimal self-reported symptom scores around the actual exacerbation.

Transitions in behaviour immediately before exacerbations were also reported in semi-structured interviews. Notably, participants reported increased app use when their symptoms were worse, as a way of refreshing their memory on self-management techniques such as breathing or relaxation exercises. This was also true of those who had more mild symptoms and had yet to experience an exacerbation, who believed they would use the app more when necessary.

*“when I do get worse I’ll use it a lot more I think.” [P6-male]*

Conversely, others instead said that they use the app less when they are particularly unwell, as they do not have the capacity to engage with it.

*“if I need my salamol I don’t even think about it. It doesn’t, it doesn’t even occur to me to write that down or record it” [P1-male]*

Despite several users becoming more engaged immediately prior to an exacerbation there is no strong evidence that this increased engagement remains short-term after the exacerbation. Figure 1 (right) shows the distribution of app use in a 70-day window post exacerbation. The shading represents the original groupings (i.e., in the 70-days prior) with the histogram being stacked so the overall area matches the left panel. We note a slight increase of infrequent users (16.7%) post exacerbation. For 9% of exacerbations there is either a notable gap in self-reports directly after exacerbation, and/or a registered symptom score of 4 (i.e., needed to seek emergency care) highlighting possible disengagement due to a deteriorated condition.

### Users’ confidence in recognising risk

A key theme from user interviews was a lack of confidence around exacerbations and how to identify one. Particularly, the difficultly to differentiate between an exacerbation, a heavy cold, a chest infection, or otherwise was discussed.

*“if that’s what an exacerbation is, i.e., it’s just a chest infection. Or does it mean that, I don’t know, it’s difficulty breathing and you need to take the inhaler? So I don’t know what it is no” [P1-male]*

This was especially true for those who also suffer from other health complications, such as asthma or bronchiectasis. Participants noted that sputum changes are not always a reliable indicator.

*“I had two exacerbations, late last year, both hospitalised and I didn’t have the normal triggers that you’d have with changes, like increased volume, coughing and things like that”*

A key barrier identified was a lack of explanation from health care professionals (HCPs), with most asserting that they had never had it explained to them.

*“That is all you hear is an exacerbation. You’re not actually told what it is. Well, they haven’t in my circumstances. Yes, it, you know, the nurses said ‘Ohh, it’s an exacerbation’ but it doesn’t explain what it actually is.” [P3–female]*

Moreover, issues accessing HCPs means that myCOPD users had minimal opportunities to clarify or ask questions. Issues accessing HCPs also led to hesitancy about medication adherence (Supplementary Note 4).

*“trying to contact your GP is, well I can’t think of a similarity but I could probably get in contact with Madonna better or more easily” [P4–male]*

**Fig. 3:**
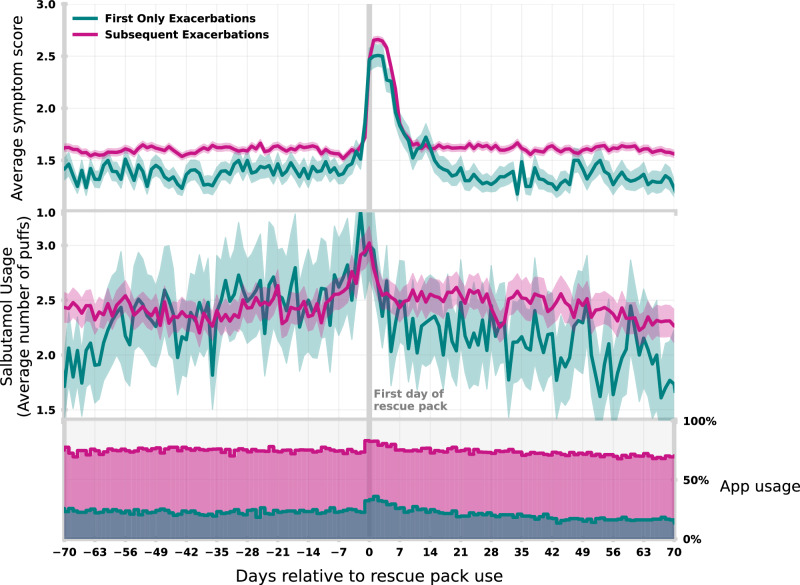
Symptom and medication reporting around exacerbations. (Top) Average daily symptom scores for 70 days prior/post to a rescue pack use for those registering their first use (blue) and those registering their second or further use (purple). (Middle) Average daily reported salbutamol usage. (Bottom) Daily app usage percentage.

Confidence in identifying risk was also reflected in self-reported data. Figure 3 compares self-reported symptom scores and salbutamol use for those registering their first exacerbation in-app relative to those reporting exacerbations having experienced one before (i.e., ‘Subsequent’). Those registering their first exacerbation consistently report lower average symptom scores (top panel; chi-square statistic=726.9, *P* = 3.05 × 10^−157^) demonstrating that users with previous experience of an exacerbation are more likely to be aware of their symptoms and report them in future events. As users increase confidence in recognising their symptoms, they also engage more frequently with the app in the longer-term (bottom panel). Users experiencing their first exacerbation also typically report lower salbutamol usage (middle panel). Salbutamol (classified as a SABA) is commonly used for immediate relief of symptoms including coughing, wheezing, and breathlessness. Increased usage reflects that the individual has experienced more breathlessness through a given day and may be indicative of a more acute condition. Regardless of experience, peak salbutamol use occurs on the first day of the exacerbation whereas average symptom scores peak days later. This indicates users self-report a deterioration through medication before typically self-recognising the deterioration in symptom scores.

Higher engagement for more experienced users is also found when considering the proportion of frequent, intermediate, and infrequent users by GOLD group (Supplementary Fig. 7). The proportion of users with a history of exacerbations (C and D) increases with engagement, reflecting that users are more likely to engage as their condition becomes more of a burden to self-manage and confidence to identify risk increases with experience of previous exacerbations.

### Disease acuity and engagement

**Fig. 4:**
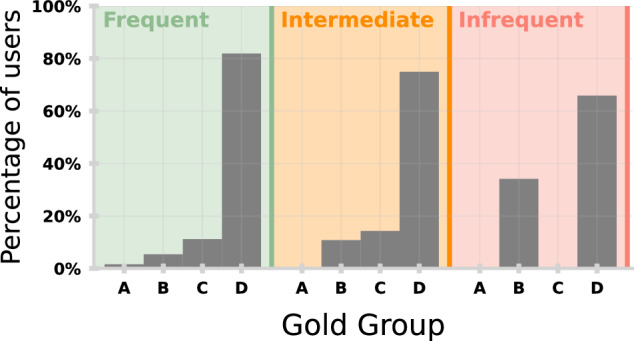
Distribution of myCOPD users stratified by GOLD group and engagement. Percentage of users in each GOLD group for infrequent (red), intermediate (orange) and frequent (green) users.

Figure 4 shows the proportion of frequent, intermediate, and infrequent users by GOLD group. The GOLD 2022 guidelines use a combined COPD assessment approach to group patients according to exacerbation history and symptoms (Fig. 6B). Overall, the majority of users reporting exacerbations are in higher risk groups, predominately represented by group D. The proportion of users with a history of exacerbations (C and D) increases with engagement, reflecting that users are more likely to engage as their condition becomes more of a burden to self-manage and confidence to identify risk increases with experience of previous exacerbations.

### How engagement impacts machine learning

**Fig. 5:**
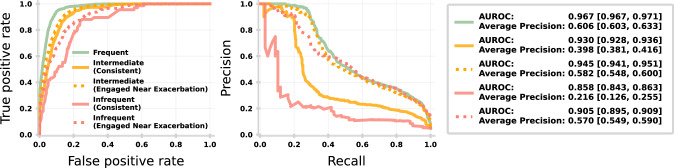
Machine learning performance for different engagement groups. (Left) ROC curve for ML model applied to frequent (green), intermediate (orange) and infrequent (red) user groups. The latter are further split by consistent engagement throughout the 70-day window prior to exacerbation (solid lines) and those becoming more engaged near (21 days prior) exacerbation (dotted lines). (Middle) Precision-recall curve for each group. (Right) AUROC and Average-Precision with 95% confidence intervals.

Figure 5 compares the performance of our XGBoost model predicting exacerbation up-to three-days in advance. Model performance, measured by AUROC and AUPR, has been computed from the hold-out test sets of simulated exacerbations for each of the following user groups (darker shaded in Fig. 2): Frequent, Intermediate (Consistent), Intermediate (Engaged Near Exacerbation), Infrequent (Consistent), Infrequent (Engaged Near Exacerbation). Predictions are made daily per user (from 55 days before to 70 days post exacerbation) and exacerbation is the positive class. Performance should only be used for contrastive purposes due to simulation of self-reported symptom scores (see Methods).

Both AUROC and average precision improve with 70-day engagement (i.e., infrequent to frequent), however, for transitional users (Engaged Near Exacerbation) the drop in performance relative to frequent users is minimal. This demonstrates that transitional engagement is more important for the safety of ML models than increasing overall engagement (i.e., regardless of current condition).

## Discussion

In this study, we classify user engagement with a self-management app, identify barriers to engagement through interviews and focus groups, and directly quantify how this could impact the safety of a ML model. 60.3% of users engage and self-report information frequently. As predicted by the literature^17^, perceived usefulness is a good indicator of app usage and adherence to self-reporting. Figure 3 demonstrates that a history of exacerbations lead to markedly higher app engagement as disease burden comes to the forefront in the individual’s day-to-day life. This further demonstrated in Fig. 4 with individuals in higher risk categories typically showing more engagement. This is more nuanced than user monitoring symptoms, instead exploiting the affordances of the app to manage medication. Despite this, adherence is not consistent. App usage increases immediately around exacerbations (Fig. 3), despite users highlighting a lack of clarity about what exacerbation means.

For predictive ML models, it is critical that users report frequently and accurately. However, the human-AI partnership must be carefully balanced to achieve useful data while not subjecting the user to burdensome self-reporting behaviours and possible desensitisation^18^. Figure 5 clearly demonstrates that transitional behaviour (i.e., becoming more engaged around exacerbations) should be a more important focus than increasing overall engagement. Encouraging users to engage when they are starting to feel unwell will not only benefit the ML model, but potentially help the user see the long-term utility of the app and reduce treatment burden.

The early identification of exacerbations is key for COPD patients to prevent or treat them with medication^19^. Figure 3 shows a ‘time-lag’ where use of reliever medications begins to increase before the self-reported symptom scores. Self-recognition of a decline in health appears to be delayed restricting algorithmic ability to make timely predictions from self-reported scores alone. This suggests apps should prompt users to review and report their condition when an uptick of reliever medications is identified. Despite this, COPD patients report challenges in recognising what an exacerbation specifically is. Research suggests that only around 60% of exacerbations are reported to healthcare professionals, suggesting that they often go unidentified and possibly untreated^20^. This was supported by our qualitative analysis with users uncertain of what to expect during an exacerbation or misinterpreting symptoms as indicators of other, related conditions. Despite this, users should be encouraged to report abrupt changes in condition, even if they do not understand the specifics. Users noted further concerns and hesitancies around taking medications in interviews (see Supplementary Note 4). Inability to discuss side-effects and medication purpose with HCPs may discourage users to adhere to prescribed treatment.

Strengths of this study include the volume of data collected by myCOPD over five years; identification of a clean sample of exacerbations from rescue-pack medication usage; combination of qualitative and quantitative approaches to articulate the balance between human orientated goals and data requirements of ML models. Limitations of this study include inclusion criteria which likely target more engaged users overall than average (i.e., those who report medications and engage with interviews); however, multiple engagement groups demonstrate diversity in the cohort. Another limitation includes the reliability of the self-reported symptom scores. As shown in Fig. 2, a significant fraction of users report minimal variability in their scores, the impact of which is not considered in our predictive models. Models predicting short-term exacerbations would benefit from a variety of self-reported data including activity and physiological measures from smart devices, oxygen therapy and dietary information.

Our research has implications for the design and engineering of mHealth apps, along with how the public should be encouraged to use it. It is critical that the developers of mHealth apps validate predictive models and JITAIs for different levels of engagement to determine safe conditions for its usage. For ML models predicting short-term risk, users of mHealth should be particularly encouraged to engage when they notice changes in their condition. This likely provides the most predictive data for ML models to maximise digital safety while minimising treatment burden on the individual and risk of disengagement through desensitisation.

## Methods

### Self-reported in-app data

**Fig. 6:**
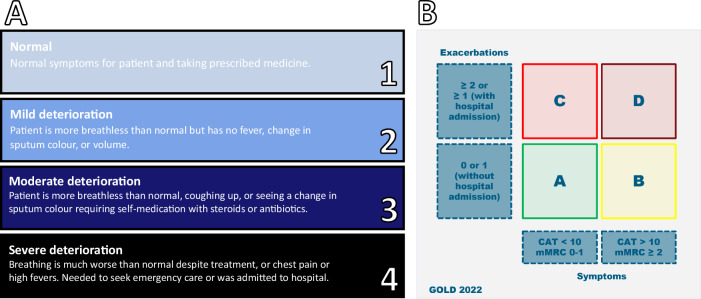
Metrics for COPD condition. **A** 4-point scale for daily self-assessed symptom scores registered in myCOPD, **B** GOLD 2022 groups.
- **COPD Assessment Tests** (CAT): a validated instrument quantifying the long-term disease burden of COPD^21,22^. Evaluated approximately monthly, the CAT is an eight-question assessment indicating the impact of COPD on a user’s overall health^23^.
- **Prescribed and reliever medications** taken for the treatment of COPD. This included routine medications (e.g., Muscarinic-Antagonists, Long-Acting Beta-Agonists, Inhaled Steroids), along with reliever medications (e.g., Short-Acting Beta-Agonists (SABAs), Rescue Packs) taken as an immediate response to a self-identified worsening of condition.
- **Exacerbation history** reported annually. Along with CAT this is used to compute long-term acuity of condition as defined by the Global Initiative for Chronic Obstructive Lung Disease (GOLD) criteria^24^. The GOLD 2022 guidelines use a combined COPD assessment approach to group patients according to exacerbation history and symptoms (Fig. 6B).

We retrospectively evaluated self-reported data from users of myCOPD between January 1st, 2017, and October 3rd, 2022. All users of myCOPD are clinically diagnosed with COPD, with usage limited to patients “prescribed” the app by clinicians as part of agreed care plans. myCOPD facilitates self-management of COPD through providing educational content, pulmonary rehabilitation, localised weather/pollution levels, and digital diaries for users to keep track of medications, symptoms, and exacerbations. Further information on myCOPD and data collection can be found in the Supplementary Material Note 1. Self-reported information included:

- **Daily self-assessed symptom scores** prompted on app opening ranked on a 4-point scale (Fig. 6A). Symptom score reporting represents a simplistic but high completeness data source (relative to other data collected in myCOPD). To make short-term predictions of risk, it is critical to include data which is updated frequently.

**Fig. 7:**
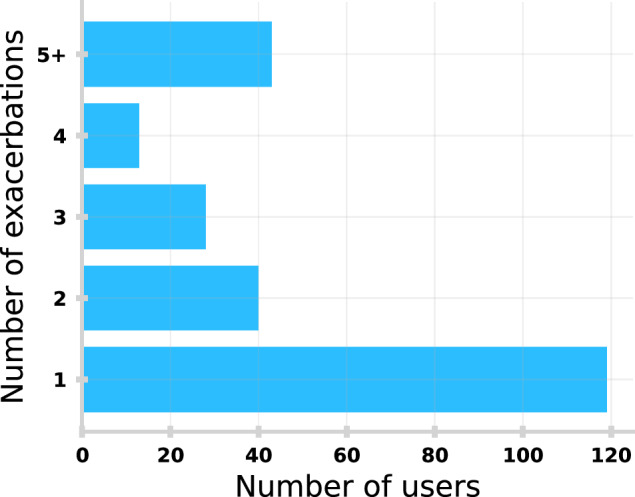
Distribution of exacerbations across cohort. Number of users (*n* = 243) in study cohort stratified by the number of exacerbations (*n* = 727) represented.

Users also provided basic demographic (e.g., age, sex, postcode) and lifestyle information (e.g., smoking status) along with other clinically validated assessment scores (Modified Medical Research Council Dyspnoea scale). To investigate app usage around exacerbations we identified users who registered the use of a ‘Rescue Pack‘ in their medication diaries (i.e., short course of oral steroids (Prednisolone) and antibiotics (e.g., Amoxicillin, Doxycycline) taken as a response to deteriorating symptoms as part of their acute exacerbation plan^25^). We did not include longer courses ( > 10 days) to avoid including weaning/maintenance prescriptions. This resulted in 727 exacerbations by 243 unique users (Age: μ = 68.8, σ = 8.3; Sex: 60.7% Male, 39.3% Female) who were registered throughout the study period. Figure 7 shows the distribution of exacerbations across the total cohort. Our selection criteria are strict to ensure we are selecting a clean sample of exacerbations with a well-defined start date, however, naturally selects users with higher disease acuity (i.e., having been prescribed a home-use Rescue Pack). We have quantified this difference in acuity in Supplementary Fig. 1. Despite this we find the selected cohort exhibit similar characteristics to the overall userbase (e.g., Age of all users: μ = 68.4, σ = 10.9).

The study received ethics approval from the University of Southampton’s Faculty of Engineering and Physical Science Research Ethics Committee (ERGO/FEPS/66535) and was reviewed by the University of Southampton Data Protection Impact Assessment panel, with the decision to support the research.

### Predicting exacerbations with machine learning

Our ML model is derived from previous work outlined in Chmiel et al. (2022) where a gradient-boosted decision-tree algorithm (XGBoost^26^) predicted exacerbations up-to three days in advance from self-reported data in myCOPD. Gradient boosted trees are examples of boosting algorithms which aim to combine an ensemble of weak learners in order to decrease bias whilst preserving or lowering variance in the prediction error, making them typically more desirable over other tree-based algorithms. A three-day window was chosen, based on clinical guidance to enable pre-emptive actions for the user while ensuring the exacerbation could be reliably predicted. Here, we aimed to quantify how app engagement and self-reported data quality impacted the performance of this algorithmic approach.

We stratified each user group by engagement and reporting quality, and then validated the performance of a model using only features relating to self-assessed symptom scores (Table 1). In Chmiel et al. (2022) predictive features were generated from a range of sources (e.g., symptom scores, CAT, demographics), however, in this study we only use symptom scores to ensure differences in model performance are related to engagement and reporting differences. An importance plot of the chosen features is provided in Supplementary Fig. 3.

**Table 1:** Summary of input features for models

Feature	Description	Time-Period
Mean score	Mean symptom score in time-period	(4-days, 8-days, 15-days)
Standard Deviation in scores	Standard deviation in symptom scores in time-period	(4-days, 8-days, 15-days)
Count	Number of registered symptom scores in time-period	(4-days, 8-days, 15-days)
Minimum score	Minimum symptom score registered in time-period	(4-days, 8-days, 15-days)
Maximum score	Maximum symptom score registered in time-period	(4-days, 8-days, 15-days)

Variances in performance also result from under/over representation of user groups in the training set (i.e., bias). To normalise, we simulated 1000 exacerbations for each user group creating empirical distributions fit to average symptom score values and frequencies from reporting prior to the real exacerbations of users in this study. Each simulated exacerbation was then sampled directly from the empirical distributions for reporting frequency and score to create complete series from 70 days prior to 70 days post exacerbation. Data was split 75–25 into train and hold-out test sets split at the series level (i.e., a simulated exacerbation appears exclusively in train or test). A binary prediction (of exacerbation in the next three-days) was generated for each day from 55 days prior (ensuring 15-day features are complete) to 70 days post exacerbation. Predictions made during the exacerbation were excluded. The XGBoost model was trained by 5-fold and grouped cross-validation grouped at the series level. Model hyperparameters were found using out-of-fold validation samples by Bayesian optimisation via the Tree-Structured Parzen Estimator (Optuna^27^). The best model hyperparameters can be found in Supplementary Table 1.

Model performance was estimated through area-under the receiving-operator characteristic curve (AUROC) and area-under the precision-recall curve (AUPR). Due to class imbalance (positive class fraction: 0.04) average precision is considered the key performance metric. We note our approach is designed to contrast impact to model performance from user engagement only and AUROC/AUPR scores are not indicative of performance in practice. We also perform this analysis using a logistic regression model (Supplementary Note 3) to justify the selection of XGBoost and confirm the differential performance trends are consistent.

### Qualitative data

Qualitative data were collected to triangulate with the quantitative data during the analysis stage. A mixed-methods design was chosen to understand both the impact of engagement with myCOPD on the machine learning models, and also the subsequent experience of app users receiving the risk prediction. Qualitative exploration of subjective experiences of app users aimed to offer explanations for engagement data, providing context for the objective quantitative data which evidences self-reported experiences at scale.

**Fig. 8:**
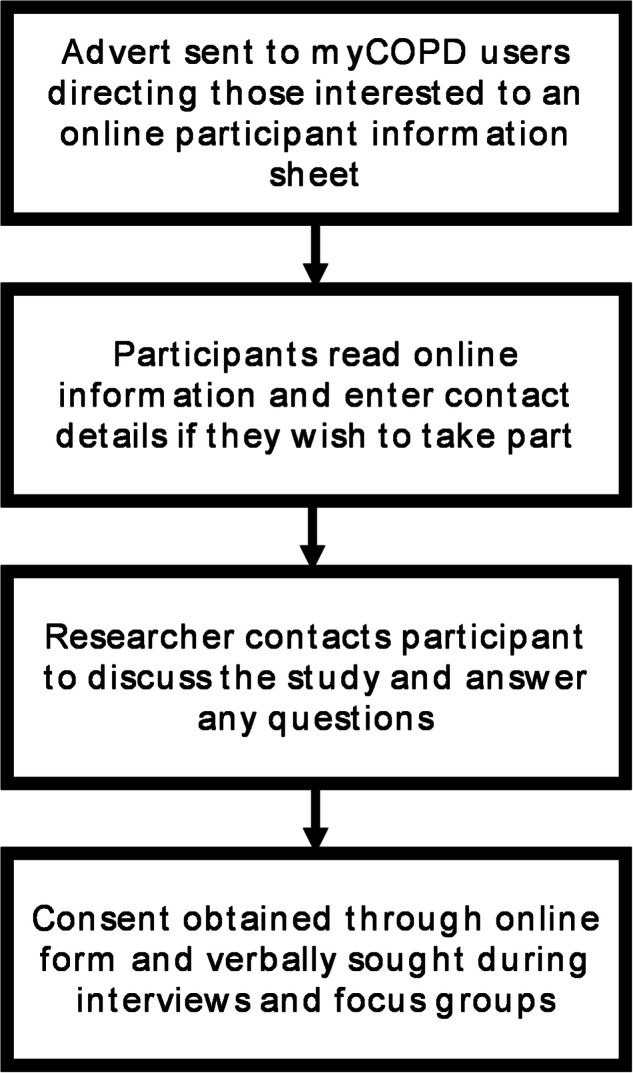
Recruitment process for qualitative interviews and focus groups. Flow diagram representing the stages associated with recruitment for qualitative research.

Qualitative data were obtained through semi-structured interviews (*N* = 7) and focus groups (*N* = 8) with myCOPD users held online (via phone/video call) in 2022, recruited and consented through myCOPD (Fig. 8). Those recruited were advised to contact the research team via email to discuss participation, directed to an online consent form, and entered their contact details on an online platform (hosted on Qualtrics) containing the participant information sheet. Participants were each paid £25 for their time. Individuals were eligible to participate if they were (1) aged 18 and above and (2) had a diagnosis of COPD. There were no other exclusion criteria.

Sample size was determined using an information power approach, whereby the level of information provided by these 15 participants was sufficiently detailed and rich to address the research questions, particularly given the specific study population and aims. Interviews and focus groups were conducted by an experienced qualitative researcher, using a topic guide developed by the researchers and study stakeholders to address the study aims. Questions focused on participants’ experience of using myCOPD, their understanding of exacerbations, and how they may perceive getting information regarding exacerbation risk generated from machine learning. B.C. transcribed recordings of the interviews/groups as the first stage of analysis. Thematic analysis was performed on the data in accordance with the six steps outlined by Braun and Clarke^28^. Transcripts were coded inductively by B.C., and the codes were developed into themes to present shared meaning within the data. Codes and themes were discussed with the research team (B.A., B.P.), who independently checked transcripts to ensure that the themes were representative of the data. The qualitative data collection received ethical approval from the University of Bath Psychology Research Ethics Committee [ref 22–041].

### Reporting summary

Further information on research design is available in the Nature Research Reporting Summary linked to this article.

### Supplementary information

Supplementary Material

Reporting Summary

## Data Availability

Aggregated data will be made available upon reasonable request to persons with a university affiliation. Requestors will need appropriate data protection, governance, and ethical review in place. Please contact C.J.Duckworth@soton.ac.uk for quantitative data enquiries and b.cliffe@westminster.ac.uk for qualitative data enquiries.

## Code availability

Quantitative analysis and modelling was performed in Python v3.8.12 and made use of the following packages: numpy, pandas, sklearn, optuna, XGBoost, matplotlib. Code will be made available upon reasonable request. Please contact C.J.Duckworth@soton.ac.uk.
