# Supplementary material for: Characterising user engagement with mHealth for chronic disease self-management and impact on machine learning performance
Source: NPJ Digit Med. 2024 Mar 12;7:66. doi: 10.1038/s41746-024-01063-2 (PMC10933254; doi:10.1038/s41746-024-01063-2)
Supplement: Supplementary file 1 — Supplementary Material [file 41746_2024_1063_MOESM1_ESM.docx]

**Supplementary Material**

**Supplementary Note 1: myCOPD and In-App Data**

myCOPD was developed by my mHealth, who are a team of doctors, professors, nurses, researchers, technologists, and pharmaceuticals experts and has been externally peer- reviewed by NHS consultants. my mHealth is a UK based company founded by NHS respiratory consultants and is one of four long term condition apps including myDiabetes and myAsthma. The apps can be accessed using any internet connected device and by download from the Apple or Google play app store. myCOPD has been adopted by over 70% Integrated Care Boards across England, Scotland, and Wales and has been in use within the NHS since 2016. myCOPD is a class 1 MHRA accredited medical device that has received approvals for UKCA marking, DSPT, DTAC and Cyber Essentials Plus. Clinically the app is DTAC and DSPT compliant and holds a DCB0129 folder for sharing with user groups. The company is also QISMET accredited, which whilst primarily focusses on diabetes, provides some measure of quality.

myCOPD can form part of a normal care plan for a COPD patient, with the app designed to support their self-management on a day-to-day basis. myCOPD provides digital disease specific support to thousands of users through self-management plans, comprehensive education on lifestyle, device instruction (inhalers), PR, symptom, COPD Assessment Test (CAT) score and exacerbation recording, and the ability to map health trends so the user and HCPs can view health deterioration or improvement over time.

myCOPD data is stored within Amazon Web Services (AWS) London Regions only, where this has been entered into the service. Electronic data transferred to AWS is encrypted in transit and at rest. my mhealth have embedded management systems in place to ensure the security and quality of its systems and the data within. All data collected, processed and stored is done so utilising AES-256 encryption in transit and at rest. The transfer of data is via network only Transfer layer Security (TLS) 1.2 only. This includes the transmission of data from the my mhealth interface to the back up and system host (AWS) remote access to infrastructure holding patient data which is monitored on a daily basis and the company complies with the requirements for the DSPT and the DCB 0129.

The study data was originally collected by my mHealth using myCOPD. Consent for the original data collection is held by my mHealth, and the app users (the data subjects) would expect their data to be used for ethically approved research. The my mHealth Privacy Notice contains the wording: “Healthcare & research teams. This will always be anonymised unless you agree, at the time, to participate in trials using your identifiable information”. This study was conducted as secondary data analysis by the University of Southampton with the legal basis to process special category personal data in accordance with UK GDPR Article 6 (e) “Public Task” and UK GDPR Article (h) “Health or social care (with a basis in law)” and within scope of Data Protection Act 2018 Article 2 “Health or social care purposes”.

In Supplementary Figure 1 we consider the distribution of the selected study cohort across GOLD groups (Figure 1B) relative to all users of myCOPD.


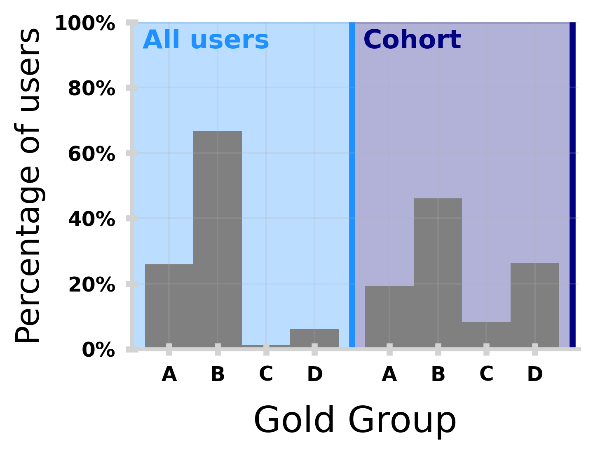


Supplementary Figure 1: Percentage of users in each GOLD group for all users on the myCOPD app and selected cohort analysed in this study.

**Supplementary Note 2: XGBoost**

Supplementary Table 1 provides the best model hyperparameters found from hyperparameter tuning. Due to class imbalance (positive class fraction: 0.04), the XGBoost model was also tested with an option to set ‘scale_pos_weight’ (i.e., hyperparameter designed for class imbalance) during tuning, however, resulted in lower performance for the positive class so was not included. Supplementary Figure 2 provides a feature importance ranking for the predictive model. Supplementary Figure 3 provides a confusion matrix of the fiducial XGBoost model dichotomised at a recall threshold of 0.9.

| Hyperparameter | Value | Search Range |
| --- | --- | --- |
| n_estimators | 250 | 25-500 |
| max_depth | 5 | 1-5 |
| learning_rate | 0.0925 | 0.001-1 |
| subsample | 0.916 | 0.1-1 |
| colsample_bytree | 0.750 | 0.1-1 |
| max_delta_step | 2 | 0-5 |
| min_child_weight | 2 | 0-5 |
| gamma | 2 | 0-5 |

Supplementary Table 1: Best hyperparameters for the fiducial XGBoost model and search range found using Bayesian Optimisation via the Tree-Structured Parzen Estimator using the Optuna library.


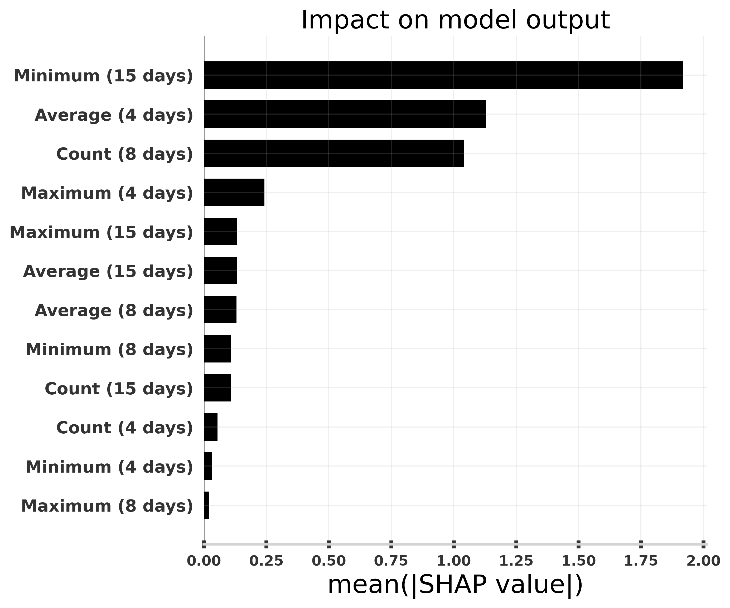


Supplementary Figure 2: Importance ranking of input features for the fiducial XGBoost model presented in the main text. Importance is estimated by the the TreeExplainer algorithm as implemented in the SHAP (SHapley Additive exPlanations) library. TreeExplainer efficiently calculates so called Shapley (SHAP) values, a game theoretic approach for attributing payout between coalitional players of a game. In the context of machine learning, SHAP values amount to the marginal contribution (i.e. change to the model prediction) of a feature amongst all possible coalitions (i.e. combinations of features). A higher mean SHAP value therefore corresponds to a higher feature importance.


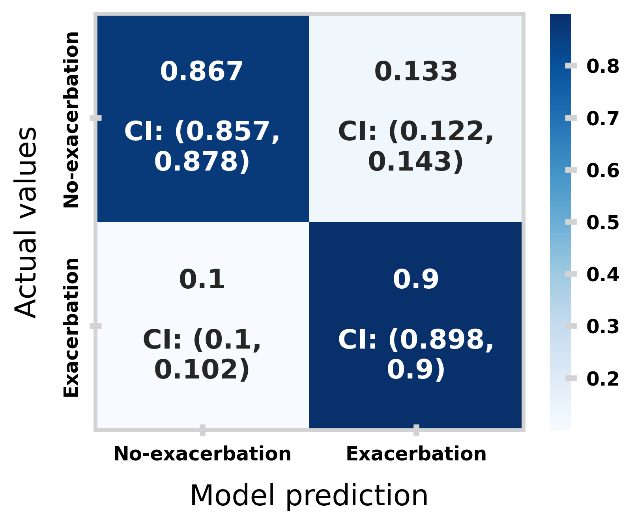


Supplementary Figure 3: Normalised confusion matrix for the fiducial XGBoost model presented in the main text. Predictions have been dichotomised at a recall threshold of 0.9, representing the requirement of a high threshold for safety, however, balancing false-alarm rates and potential disengagement. Each panel shows the 2.5%, 97.5% confidence intervals on presented values, evaluated using bootstrapping.

**Supplementary Note 3: Logistic Regression**

To justify the selection of a gradient-boosted decision-tree approach we also evaluated the predictive performance of a logistic regression model. Logistic regression, despite its name, is a linear model which aims to classify binary outcomes through probabilities estimated by the logistic function. We implement our logistic regression model using Scikit-learn, optimized for ≤300 iterations using a L2 penalty with class weights inversely proportional to class frequencies. Supplementary Figure 4 is equivalent to Figure 4 in the main text, however, evaluated for the logistic regression model. Supplementary Figure 5 provides the confusion matrix for the logistic regression dichotomised at recall = 0.9 (in parallel with Supplementary Figure 4 for the XGBoost model).

We note that the XGBoost model significantly outperforms the logistic regression for each user group and performance metric. This highlights the requirement for a model capturing non-linear relationships. The differential performance between user groups of the logistic regression model also reflects the general trends found with the fiducial XGBoost model (e.g., performance remains high for transitional users).


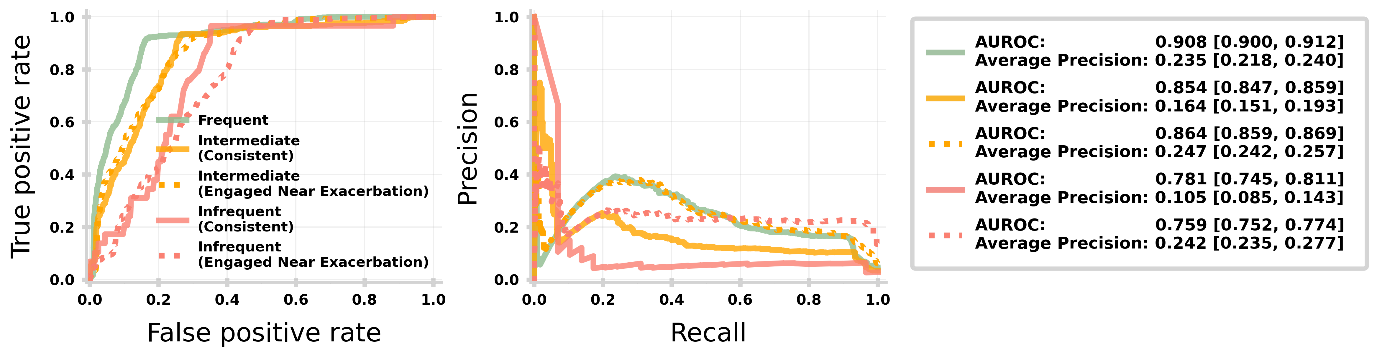
*Supplementary Figure 4: (Left) ROC curve for Logistic Regression model applied to frequent (green), intermediate (orange) and infrequent (red) user groups. The latter are further split by consistent engagement throughout the 70-day window prior to exacerbation (solid lines) and those becoming more engaged near (21 days prior) exacerbation (dotted lines). (Middle) Precision-recall curve for each group. (Right) AUROC and Average-Precision with 95% confidence intervals. This replicates the trends*


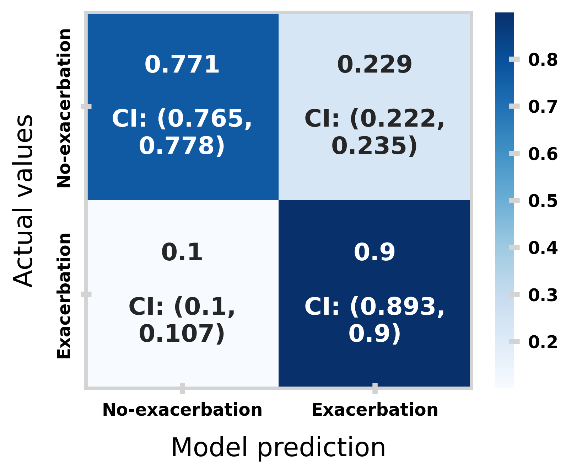


Supplementary Figure 5: Normalised confusion matrix for the logistic regression model. Predictions have been dichotomised at a recall threshold of 0.9, representing the requirement of a high threshold for safety, however, balancing false-alarm rates and potential disengagement. Each panel shows the 2.5%, 97.5% confidence intervals on presented values, evaluated using bootstrapping.

**Supplementary Note 4:** **Concerns and hesitancies around taking medications**

Further to difficulties in identifying exacerbations, barriers to self-managing extended to the hesitancies some COPD patients felt around taking medication. Whilst some participants reported an absolute willingness to take medication when needed, others discussed their concerns around possible side effects.

“I've had side effects which have been, sort of, they put you off. There was one, I forget what it was called, but one of the inhalers I got with the steroids in. It gave me terrible cramp. I used to get cramp all the time, so I just stopped using it” [P2 – male]

This participant exemplified how the side effects were significant enough for them to cease medication use. Others reported abstaining from taking medication due to the overwhelming amount they needed, for example one participant discussed this in relation to their rescue pack.

“I just don’t like the idea of shoving 6 tablets into myself for 6 days” [P4 – male]

Another participant felt similar and noted that a lack of understanding around the purpose of the various medications contributed to this hesitancy.

“I got to the stage where because I take that much, I got to the stage of stopping it all because I was just so fed up and I was sick of these medications I didn't know what I was taking it for. I stopped, which I shouldn't have done because I ended up in hospital. And, you know, but if it was explained to you why you were taking it what it's for, you know, I know they put the leaflet in, but nobody reads those leaflets” [P3 – female]

This participant elaborated on a previous negative experience they had due to a medication conflict that was not identified

“You’re given medications but you’re not told what the medication’s for. You take it and then find out that you shouldn't have been taking it. Cause of, say, like, I've got kidney disease and one of the medications were to take because of kidney disease.” [P3 – female]

This increased their anxiety around taking medications and their possible side effects. However, whilst some participants wanted more input from HCPs to help understand medications, others shared a distrust of HCPs which made them wary of prescriptions. This distrust stemmed from queries around the intentions behind prescription changes

“sometimes they seem to be coming at it from the wrong angle, instead of doing what's best for me, they do what’s best for the practice rather than what's best for me... And it's not just me who's on this experience. I know where my wife like, she's had the similar experience where they've sort of tried to change medication mainly on the best basis of the financial side” [P2 – male]

As a consequence, this participant mentioned that they instead try to self-manage with exercise or over-the-counter remedies instead

“when the wheezing started coming on, I'll, I'd try a few fisherman's friends first, try and stop the wheezing” [P2 – male]

This could mean that there may be a delay between an individuals’ symptoms flaring up and reporting rescue pack use.

**Supplementary Note 5: Study Protocol**

**Protocol: A mixed methods approach to characterise user engagement with mHealth for chronic disease self-management and how engagement impacts predictive machine learning using self-reported data**

**Background**

Self-management is a key tenet of chronic diseases such as Chronic Obstructive Pulmonary Disease (COPD), whereby patients hold some responsibility for the day-to-day management of their symptoms and condition (British Lung Foundation, 2021). Technology, such as mobile health, can facilitate self-management for example through providing a space for patients to monitor symptoms and receive health education (Slevin et al., 2019; Knox et al., 2021). Incorporating machine learning could improve mobile health by using patients’ self-reported data to provide them with useful information regarding their condition to improve their self-management. For example, it could alert patients if data suggests they are at a greater health risk or suggest what they may need to reduce their risk (Chmiel et al., 2022). However, for this to be successful and accurate, we first must understand more about self-management via mobile healthcare apps and how this may influence a machine learning model.

To explore this, we will look at app users and data from one particular app: myCOPD. myCOPD is an NHS approved self-management app for COPD patients in which they can monitor symptoms, log medication use, watch instructional/educational videos, and learn about self-management techniques.

**Aims**

1. To classify user engagement with a self-management app for Chronic Obstructive Pulmonary Disease (COPD)
2. Explore motivations for and challenges to engagement
3. Understand how levels of engagement may impact the accuracy of a machine learning model for predicting health risks.

**Methods**

This will be a mixed-methods study combining quantitative data science and qualitative methods, where data will be mixed during the analysis stage using a triangulation design (Creswell & Plano Clark, 2007). The motivation for a mixed-methods design arises from the need to understand the impact of engagement with self-management mobile health on both the machine learning models and on the real-world app users whose health behaviours may be impacted by the outcomes. Here, the qualitative findings can help to contextualise patterns in the data with subjective accounts of app use. Equally, the quantitative data can be applied to inform engagement with app users and to validate their self-reported experiences.

*Quantitative methods*

Self-reported data from myCOPD app users will be retrospectively evaluated to explore engagement with the app. Data analysed will include symptom scores that users self-assess daily, COPD assessment tests, a standard instrument whereby users are prompted to complete every month to assess the impact of COPD, medications the user is taking (including routine medication and those taken to relieve an exacerbation), and the app user’s exacerbation history. Data will be plotted to identify patterns and to classify user groups relative to engagement levels and use around periods of worse health (i.e., COPD exacerbations).

Symptom score data will be input to an extant machine learning model to explore its performance. The model has been described elsewhere (Chmiel et al., 2022). This model was chosen as it was found to successfully use myCOPD data to predict exacerbations up-to 3 days before occurring.

*Qualitative methods*

Semi-structured interviews and focus groups will be conducted to explore subjective experiences of using myCOPD.

An information power approach will be used to determine sample size, whilst also taking practical considerations into account such as the time and resources available. An information power approach outlines that the sample required depends on the level of information that each participant can provide. In practice, this means that fewer participants are required if each participant is able to provide rich and thorough insights relative to the research questions. Due to the specific population and research question under investigation, it is anticipated that roughly 10-15 participants will be sufficient (Malterud et al., 2016).

Participants will be myCOPD users aged 18 and above who respond to an advert within the app outlining the qualitative study. There will be no other exclusion criteria. In the advert, myCOPD users will be directed to an online participant information sheet with space to enter their contact details should they wish to take part. They will then be contacted by the researcher to discuss participation. Electronic consent will be obtained via an online consent form, and verbal consent will also be sought during the interview/focus group. Sessions will be conducted virtually, either over video or telephone call, and will last roughly 30-60 minutes. All sessions will be recorded for transcription purposes. Participants will receive £25 for taking part.

Interviews and focus groups will be conducted by a researcher with expertise in qualitative methods, based on a set of broad questions (by definition, the seed questions are typically broad-stroke, and not all will be used as the interview progresses). The questions have been developed by the researchers and are designed to address the research questions. Specifically, the interview will explore participants’ use of myCOPD, their motivations for using myCOPD, barriers and facilitators for myCOPD use, and how they may perceive getting information regarding exacerbation risk or a medication review from the app generated from machine learning. Please see the topic guide in appendix A.

Transcription will be completed by the researcher as the first step in analysing the data. A reflexive thematic analysis following Braun and Clarke’s six steps will be performed by the qualitative researcher with input from the wider team (Braun and Clarke, 2006; 2019). Following transcription, the transcripts will be coded inductively. Codes will be discussed with the team to explore possible interpretations. Codes will then be organised into themes relative to shared meaning underpinning the data. Themes will also be discussed with the wider team, and they will be checked against the transcriptions until it is agreed that they are representative of the dataset.

**Results and Discussion**

The quantitative and qualitative findings will be triangulated, as described above, to inform the analysis and reporting of findings. The results will be written in a mixed-methods paper and submitted to a journal appropriate for the methods and topic, such as a digital health journal.

These findings will contribute to our understanding of engagement with self-management mobile health and its potential impact on machine learning models and their capabilities in prediction health risk, and the possible real-world impact on app users. This could have implications for the development of technologies incorporating these methods to improve the self-management of chronic health conditions.

**References**

British Lung Foundation. (2021, March 9). *Your COPD self-management plan*. Asthma + Lung UK. <https://www.blf.org.uk/support-for-you/copd/your-copd-self-management-plan>

Slevin, P., Kessie, T., Cullen, J., Butler, M. W., Donnelly, S. C., & Caulfield, B. (2019). A qualitative study of chronic obstructive pulmonary disease patient perceptions of the barriers and facilitators to adopting digital health technology. *DIGITAL HEALTH*, *5*, 2055207619871729. <https://doi.org/10.1177/2055207619871729>

Knox, L., Gemine, R., Rees, S., Bowen, S., Groom, P., Taylor, D., Bond, I., Rosser, W., & Lewis, K. (2021). Using the Technology Acceptance Model to conceptualise experiences of the usability and acceptability of a self-management app (COPD.Pal®) for Chronic Obstructive Pulmonary Disease. *Health and Technology*, *11*(1), 111–117. <https://doi.org/10.1007/s12553-020-00494-7>

Creswell, J. W., & Plano Clark, V. L. (2007). *Designing and conducting mixed methods research*. SAGE Publications.

Chmiel FP, Burns DK, Pickering JB, et al. Prediction of Chronic Obstructive Pulmonary Disease Exacerbation Events by Using Patient Self-reported Data in a Digital Health App: Statistical Evaluation and Machine Learning Approach. JMIR medical informatics 2022;10(3):e26499.

Malterud, K., Siersma, V. D., & Guassora, A. D. (2016). Sample Size in Qualitative Interview Studies: Guided by Information Power. *Qualitative Health Research*, *26*(13), 1753–1760. <https://doi.org/10.1177/1049732315617444>

**Supplementary Note 5: Topic Guide**

How do you feel about entering data into the app?

- - *Would you have any concerns about this?*
  - *Are there any types of information you wouldn’t want the app to have? Why?*

How do you feel about having to use this app on a regular basis (e.g. daily?)

- *How long would you be willing to spend interacting with this app on a regular basis?*
- *What would be the barriers/challenges to using this app regularly?*

How would you feel about receiving regular notifications from the app, either reminding you to use it or providing recommendations?

- - *How often would you like to receive notifications?*
- *What sort of notifications would you be happy to receive?*

How confident would you be in a prediction about your exacerbation risk made purely by technology with no human involvement?

- - *Why?*
  - *What would increase your confidence in it?*
  - *How would this prediction from the app compare to your own awareness of your risk of exacerbating? (tapping into self-efficacy and where the app fits into this)*

How comfortable would you be acting on a recommendation given by technology in an automated process?

- *Why?*
- *What could make you more or less likely to act on a recommendation given by the app?*
- *How would receiving a recommendation from the app compare to receiving it from a clinician? (tapping into how the app fits within the traditional clinician-patient care regime)*
- *How would the app fit into any pre-existing care regime?*

If you were presented with a worrying risk assessment (i.e., told you were at higher risk of having an exacerbation), how would you react?

- - *How would this make you feel (i.e., would it make you worried/anxious/panic at all?)*
  - *Would you be motivated to engage with your self-management plan?*

How confident do you feel in self-managing?

- - *For example recognising and monitoring symptoms*
  - *Entering data into the app*
