# Supplementary material for: Characterising user engagement with mHealth for chronic disease self-management and impact on machine learning performance
Source: NPJ Digit Med. 2024 Mar 12;7:66. doi: 10.1038/s41746-024-01063-2 (PMC10933254; doi:10.1038/s41746-024-01063-2)
Supplement: Supplementary file 2 — Reporting Summary [file 41746_2024_1063_MOESM2_ESM.pdf]

nature portfolio

Corresponding author(s): Chris Duckworth
Last updated by author(s): Feb 16, 2024

Reporting Summary

Nature Portfolio wishes to improve the reproducibility of the work that we publish. This form provides structure for consistency and transparency in reporting. For further information on Nature Portfolio policies, see our Editorial Policies and the Editorial Policy Checklist.

Statistics

For all statistical analyses, confirm that the following items are present in the figure legend, table legend, main text, or Methods section.

n/a	Confirmed	
☐	☒	The exact sample size (*n*) for each experimental group/condition, given as a discrete number and unit of measurement
☒	☐	A statement on whether measurements were taken from distinct samples or whether the same sample was measured repeatedly
☐	☒	The statistical test(s) used AND whether they are one- or two-sided<br>*Only common tests should be described solely by name; describe more complex techniques in the Methods section.*
☐	☒	A description of all covariates tested
☐	☒	A description of any assumptions or corrections, such as tests of normality and adjustment for multiple comparisons
☐	☒	A full description of the statistical parameters including central tendency (e.g. means) or other basic estimates (e.g. regression coefficient) AND variation (e.g. standard deviation) or associated estimates of uncertainty (e.g. confidence intervals)
☐	☒	For null hypothesis testing, the test statistic (e.g. *F*, *t*, *r*) with confidence intervals, effect sizes, degrees of freedom and *P* value noted<br>*Give P values as exact values whenever suitable.*
☒	☐	For Bayesian analysis, information on the choice of priors and Markov chain Monte Carlo settings
☒	☐	For hierarchical and complex designs, identification of the appropriate level for tests and full reporting of outcomes
☒	☐	Estimates of effect sizes (e.g. Cohen's *d*, Pearson's *r*), indicating how they were calculated

Our web collection on statistics for biologists contains articles on many of the points above.

Software and code

Policy information about availability of computer code

Data collection	Data was collected through the myCOPD app. The myCOPD code is proprietary, utilising open-source programs to support the build. The version of the platform is available from the Appstore for both iOS and android devices (React Native). Current versions: Appstore – 2.5.16, Google play – 2.5.16.
Data analysis	Quantitative analysis and modelling was performed in Python v3.8.12 and made use of the following packages: numpy, pandas, sklearn, optuna, XGBoost, matplotlib. Code will be made available upon reasonable request.

For manuscripts utilizing custom algorithms or software that are central to the research but not yet described in published literature, software must be made available to editors and reviewers. We strongly encourage code deposition in a community repository (e.g. GitHub). See the Nature Portfolio guidelines for submitting code & software for further information.

Data

Policy information about availability of data

All manuscripts must include a data availability statement. This statement should provide the following information, where applicable:

- Accession codes, unique identifiers, or web links for publicly available datasets
- A description of any restrictions on data availability
- For clinical datasets or third party data, please ensure that the statement adheres to our policy

Data will be made available upon reasonable request to persons with a university affiliation. Requestors will need appropriate data protection, governance, and ethical review in place.

Research involving human participants, their data, or biological material

Policy information about studies with human participants or human data. See also policy information about sex, gender (identity/presentation), and sexual orientation and race, ethnicity and racism.

Reporting on sex and gender	A breakdown of the quantitative study cohort into Sex has been provided in the main text (methods).
Reporting on race, ethnicity, or other socially relevant groupings	Race, ethnicity or other socially relevant groupings is not captured within myCOPD at the time of writing so it is not supported in our analysis.
Population characteristics	Basic population characteristics (age, sex, disease acuity) is provided for the quantitative study cohort in the main text, with further information about myCOPD users in the Supplementary Materials.
Recruitment	The study data was originally collected by my mHealth using myCOPD. Consent for the original data collection is held by my mHealth, and the app users (the data subjects) would expect their data to be used for ethically approved research. The my mHealth Privacy Notice contains the wording: “Healthcare & research teams. This will always be anonymised unless you agree, at the time, to participate in trials using your identifiable information”. This study was conducted as secondary data analysis by the University of Southampton with the legal basis to process special category personal data in accordance with UK GDPR Article 6 (e) “Public Task” and UK GDPR Article (h) “Health or social care (with a basis in law)” and within scope of Data Protection Act 2018 Article 2 “Health or social care purposes”.<br><br>Individuals were recruited through myCOPD for the purpose of qualitative research (interviews and focus groups). After receiving an advert, users entered contact details, had direct conversations with named researchers to answer any questions and consented through online forms. The full procedure is outlined in Figure 2.
Ethics oversight	The study received ethics approval from the University of Southampton’s Faculty of Engineering and Physical Science Research Ethics Committee (ERGO/FEPS/66535) and was reviewed by the University of Southampton Data Protection Impact Assessment panel, with the decision to support the research.<br><br>The qualitative data collection received ethical approval from the University of Bath Psychology Research Ethics Committee [ref 22-041].

Note that full information on the approval of the study protocol must also be provided in the manuscript.

Field-specific reporting

Please select the one below that is the best fit for your research. If you are not sure, read the appropriate sections before making your selection.

☐ Life sciences ☒ Behavioural & social sciences ☐ Ecological, evolutionary & environmental sciences

For a reference copy of the document with all sections, see nature.com/documents/nr-reporting-summary-flat.pdf

Behavioural & social sciences study design

All studies must disclose on these points even when the disclosure is negative.

Study description	The study involved a mixed-methods approach to classify how individuals with Chronic Obstructive Pulmonary Disease(COPD) interacted with a self-management app myCOPD, understand reasons for engagement/disengagement through interviews/focus groups, and quantify how engagement would impact the performance of a predictive machine learning model.
Research sample	Quantitative data consisted of 727 exacerbations by 243 unique users (Age: μ=68.8, σ=8.3; Sex: 60.7% Male, 39.3% Female) who were registered throughout the study period. Corresponding self-reported symptom scores were also collected around the exacerbations to classify users into engagement groups.

	Qualitative data consisted of semi-structured interviews (N=7) and focus groups (N=8) from myCOPD users who consented to the study.
Sampling strategy	For the purpose of the quantitative data, users were selected on the basis of having registered a 'Rescue Pack' use in their medication diaries (i.e., short course of oral steroids (Prednisolone) and antibiotics (e.g., Amoxicillin, Doxycycline) taken as a response to deteriorating symptoms as part of their acute exacerbation plan). Our selection criteria are strict to ensure we are selecting a clean sample of exacerbations with a well-defined start date.<br><br>Qualitative data sample size was determined using an information power approach, whereby the level of information provided by these 15 participants was sufficiently detailed and rich to address the research questions, particularly given the specific study population and aims. Interviews and focus groups were conducted by an experienced qualitative researcher, using a topic guide developed by the researchers and study stakeholders to address the study aims.
Data collection	Quantitative data was collected through routine use of the myCOPD app.
Timing	We retrospectively evaluated self-reported data from users of myCOPD between January 1st, 2017, and October 3rd, 2022.<br><br>Qualitative data was collected from September 1st, 2021 to October 1st, 2022 with all interviews and focus groups taking place in 2022.
Data exclusions	We excluded exacerbations from users who reported longer courses (>10 days) of medications associated with 'Rescue Packs' since it would be ambiguous whether this pertained to a Rescue Pack or a maintenance dose.
Non-participation	No participants dropped out.
Randomization	Users of myCOPD were classified into engagement groups (Figure 3) defined by the frequency of app use. The performance of a machine learning model was then calculated using simulated data derived from each group.

Reporting for specific materials, systems and methods

We require information from authors about some types of materials, experimental systems and methods used in many studies. Here, indicate whether each material, system or method listed is relevant to your study. If you are not sure if a list item applies to your research, read the appropriate section before selecting a response.

Materials & experimental systems

n/a	Involved in the study
☒	☐ Antibodies
☒	☐ Eukaryotic cell lines
☒	☐ Palaeontology and archaeology
☒	☐ Animals and other organisms
☒	☐ Clinical data
☒	☐ Dual use research of concern
☒	☐ Plants

Methods

n/a	Involved in the study
☒	☐ ChIP-seq
☒	☐ Flow cytometry
☒	☐ MRI-based neuroimaging

Plants

Seed stocks	*Report on the source of all seed stocks or other plant material used. If applicable, state the seed stock centre and catalogue number. If plant specimens were collected from the field, describe the collection location, date and sampling procedures.*
Novel plant genotypes	*Describe the methods by which all novel plant genotypes were produced. This includes those generated by transgenic approaches, gene editing, chemical/radiation-based mutagenesis and hybridization. For transgenic lines, describe the transformation method, the number of independent lines analyzed and the generation upon which experiments were performed. For gene-edited lines, describe the editor used, the endogenous sequence targeted for editing, the targeting guide RNA sequence (if applicable) and how the editor was applied.*
Authentication	*Describe any authentication procedures for each seed stock used or novel genotype generated. Describe any experiments used to assess the effect of a mutation and, where applicable, how potential secondary effects (e.g. second site T-DNA insertions, mosiacism, off-target gene editing) were examined.*
